# A full genome tiling array enhanced the inspection and quarantine of SARS-CoV-2

**DOI:** 10.1186/s12985-023-02000-7

**Published:** 2023-03-05

**Authors:** Runzi Qi, Gang Wang, Xu Wang, Cheng Li, Lei Huang, Weixi Xiao, Bing Shao, Chunya Zhou, Xun Ding, Feng Li, Wei Zhou

**Affiliations:** 1 Hangzhou Xiaoshan Airport Customs of the People’s Republic of China, Hangzhou, 311241 China; 2 Hangzhou International Travel Healthcare Center, Hangzhou, 310061 China; 3 Centrillion Technology (Hangzhou) Co., Ltd, Hangzhou, 310053 China; 4 Hangzhou Customs Logistics Management Center, Hangzhou, 310005 China; 5 Zhoushan International Travel Healthcare Center Clinic, Zhoushan, 316004 China; 6 Centrillion Technologies, Palo Alto, CA 94303 USA

**Keywords:** Full genome tiling array, SARS-CoV-2, Ports, Inspection and quarantine, Variation

## Abstract

As the worldwide spreading epidemic of SARS-CoV-2, quick inspection and quarantine of passengers for SARS-CoV-2 infection are essential for controlling the spread of SARS-CoV-2, especially the cross-border transmission. This study reports a SARS-CoV-2 genome sequencing method based on a re-sequencing tiling array successfully used in border inspection and quarantine. The tiling array chip has four cores, with one core of 240,000 probes dedicated to the whole genome sequencing of the SAR-CoV-2 genome. The assay protocol has been improved to reduce the detection time to within one day and can detect 96 samples in parallel. The detection accuracy has been validated. This fast and simple procedure is also of low cost and high accuracy, and it is particularly suitable for the rapid tracking of viral genetic variants in custom inspection applications. Combining these properties means this method has significant application potential in the clinical investigation and quarantine of SARS-CoV-2. We used this SARS-CoV-2 genome re-sequencing tiling array to inspect and quarantine China's entry and exit ports in the Zhejiang Province. From November 2020 to January 2022, we observed the gradual shift of SARS-CoV-2 variants from the D614G type to the Delta Variant, and then to the dominance of the Omicron variant recently, consistently with the global emergency pattern of the new SARS-CoV-2 variant.

## Introduction

The primary tool that is the most widely used viral nucleic acid detection method is the real-time PCR (RT-PCR) method [1, 2]. With its high sensitivity and specificity, RT-PCR is considered a gold standard for diagnosing Covid-19 (https://apps.who.int/iris/handle/10665/331329). But as the Covid-19 pandemic continues, mutations and virus evolution bring new challenges to the method. Not only for the possibility of mutation-associated false negative [3] but also for the lack of ability to identify mutations and variants essential for making policy decisions regarding outbreak control. Next-generation sequencing (NGS) technology is an alternative virus nucleic acid detection method. This method can obtain the virus genome sequence, and by comparing it with a reference genome sequence, mutations and haplotypes can be identified [4]. We can track the origin and evolution by identifying mutation patterns or variants of the virus. Even if the costs have come down and the difficulty of protocols has eased as a legacy of the Covid -19 pandemic, however, the NGS method is limited by its relatively high cost, time-consuming and complex experimental procedures, and bioinformatic requirements for data analysis. Therefore, using NGS sequencing in large-scale clinical detection is challenging, especially on the front lines of pandemic control, such as in customs inspection and quarantine.

Microarray technology is a rapid and high throughput molecular biology detection tool. The detection rate of the re-sequencing chip can reach 96–99%, and the consistency with Sanger sequencing can reach as high as 99.99% [5]. Wang et al. have developed a re-sequencing chip detection method for more than 100 microorganisms [6]. Guo et al. have designed a microarray for detecting SARS based on Affymetrix high-density gene chip technology [7, 8]. The RPM v.1 and PathChip were respiratory pathogen arrays designed to detect various respiratory pathogens [9, 10].

Our study uses a previously reported tiling array chip to sequence the whole genome of SARS-CoV-2 [11] so that the informative mutations can identify the variants. With the ability of rapid and inexpensive full viral genome re-sequencing, this method is expected to be a tool for mutation monitoring and virus source tracing during daily inspection and quarantine in the epidemic.

## Methods

### Workflow

**Fig. 1:**
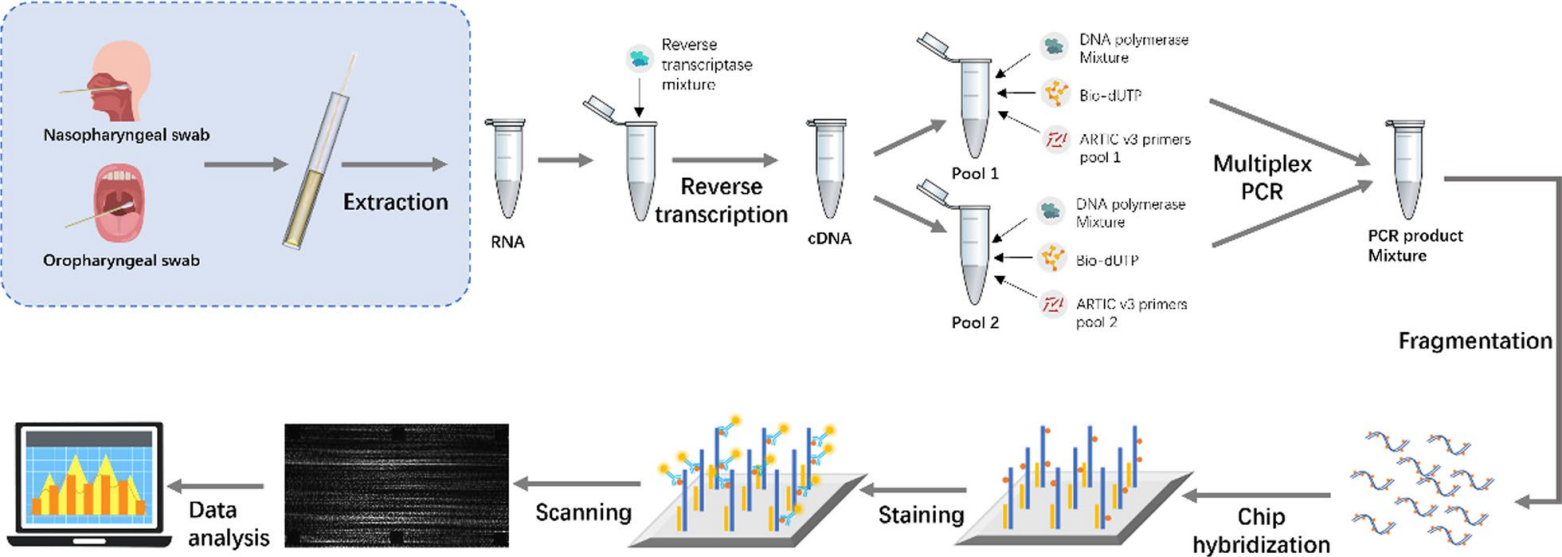
The schematic of the SARS-COV-2 re-sequencing chip testing workflow

Samples were prepared as previously described with minor modifications [11]. In brief, total RNA was extracted from the samples intercepted at the port. cDNA was prepared using HiScript® III-RT SuperMix (Vazyme) and random hexamer primer. Q5U high fidelity DNA polymerase (NEB) and the ARTIC Pool 1 and Pool 2 SARS-CoV-2 v3 primer sets were used to amplify cDNA for 35 cycles with biotin-11-dUTP (Thermo Fisher) added. DNase I was used to fragment the PCR products. The library was hybridized with the chip at 45 °C for 2 h or overnight. After hybridization, the chip was washed with Wash A and Wash B successively, stained with streptavidin-PE (Thermo Fisher) for 15 min, and washed with 4 × SSC for 5 min at room temperature. A custom-built confocal scanner was used to scan chips, and the signal intensity was obtained and used for base calling [11]. Finally, the virus genome sequence in the sample was obtained using data analysis software (Fig. 1). The specified data analysis software generated the candidate consensus sequences as FASTA files based on the scanned fluorescence intensity value from the image [11, 12]. By uploading the FASTA file onto a web application called Pangolin (Phylogenetic Assignment of Named Global Outbreak Lineages) [13], the most likely lineage to the query sequences can be assigned, and variants can be defined.

### Clinical samples

Eighty-eight RT-PCR positive samples of SARS-COV-2 were randomly selected from the entry quarantine of Hangzhou Customs Port for re-sequencing chip analysis.

6 clinical samples were selected and classified into 2 groups based on the CT values to test the chip. The 2 groups were named strong positive (SP) with CT values below 25 and weakly positive (WP) with CT values around 30. For each group, 3 samples were used as replicates.

Eighty-eight RT-PCR-positive samples were sequenced by chips to monitor the variation of imported virus cases. The FASTA files were analyzed using a pangolin web application to define the variants, especially those of concern (VOCs).

### Sanger sequencing

We checked 3 sites of the 6 positive samples by Sanger sequencing to validate the detected mutations. Primers that cover the selected sites are listed in Table 1. The extracted RNA was directly amplified using a one-step RT-PCR kit (Vazyme), and each pair of primers were used independently. The resultant PCR product was purified by a DNA extraction kit and sequenced. Sanger sequencing was completed by Tsingke Biotechnology Co., Ltd.

**Table 1:** Primers for mutation detection. Mutation positions and primer sequences are shown

Position	Sequence
C3037T	Forward: TGAGAAGTGCTCTGCCTATACAGT
	Reverse: TCATCTAACCAATCTTCTTCTTGCTCT
C14408T	Forward: TGTTGACACTGACTTAACAAAGCCT
	Reverse: TAGATTACCAGAAGCAGCGTGC
A23403G	Forward: CCAGCAACTGTTTGTGGACCTA
	Reverse: CAGCCCCTATTAAACAGCCTGC

## Result

This study used a 3 mm × 3 mm silicon-based high-density in situ synthetic tiling array chip, which contains over 250 k probes for sequencing the full genome sequence of SARS-COV-2. The testing workflow consists of two main sections. The first section is library construction, including RT-PCR (4 h) and DNA fragmentation (45 min). The second section is chip assay, including hybridization (2 h), staining (30 min), scanning (8 min per chip), and data processing (3 min per chip). The workflow goes from sample to sequence and can be finished in one day with a throughput of as high as 96 chips in parallel.

**Fig. 2:**
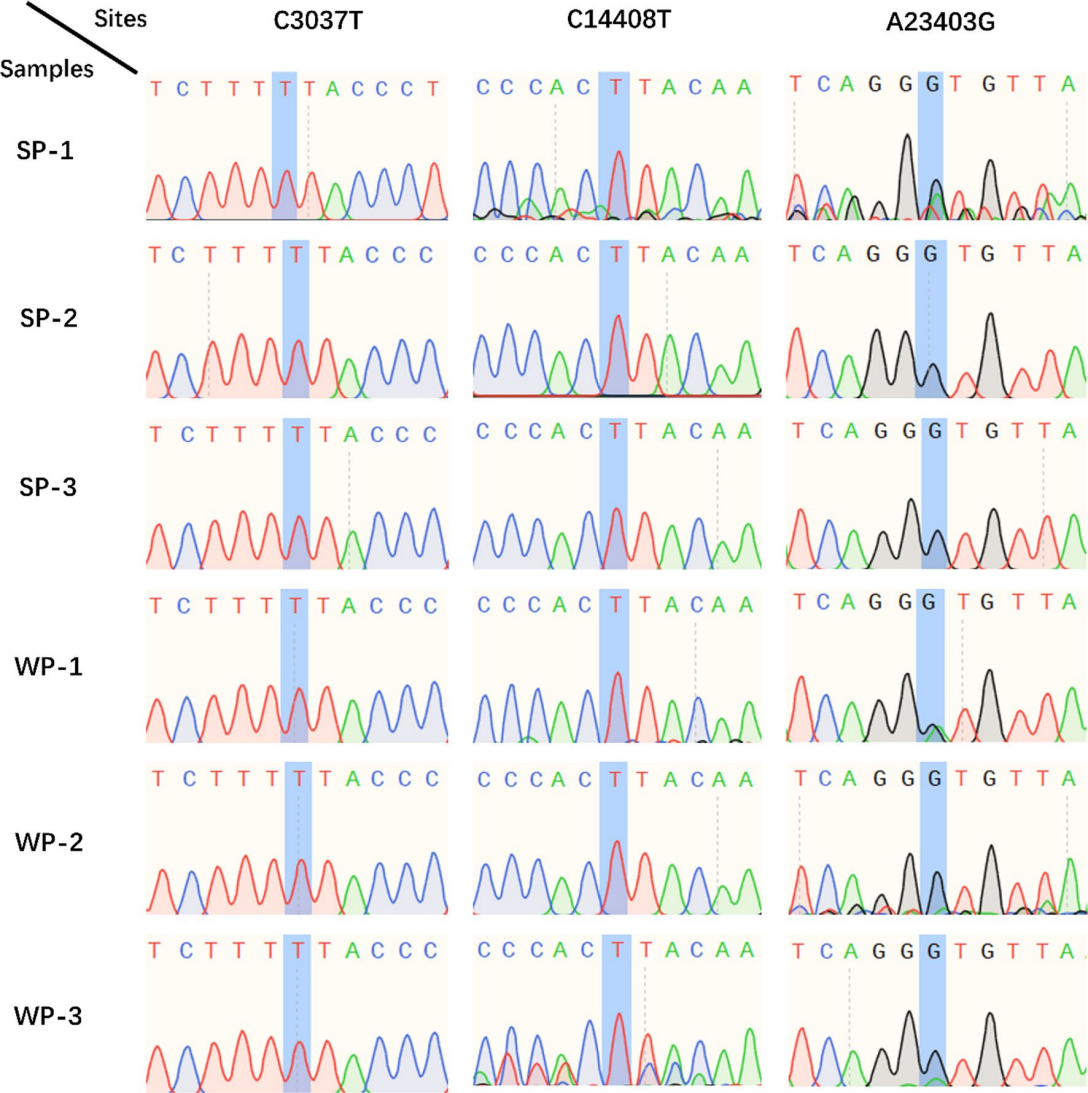
The Sanger sequencing results of the 3 selected sites of 6 samples. The mutation bases were colored in light blue

The next-generation sequencing (NGS) method was used in the previous study to verify base calling accuracy. The results indicated that the average accuracy of chip-based re-sequencing could be greater than 99.9% over 95% of ~ 30,000 bases SARS-CoV-2 genome [11]. To further validate the assay and certificate the detected mutations, 6 clinical samples with different Ct values were tested. All these samples successfully detected the whole SARS-CoV-2 genomes with relatively high coverages ranging from 99.68 to 99.96% (Table 2). Three mutations that existed in all 6 samples were confirmed by Sanger sequencing. The result showed that all these mutations called by our tiling array were consistent with Sanger sequencing (Fig. 2).

**Table 2:** Tiling array sequencing results of 6 clinical samples

Group	Samples	CT value	Coverage	Detected mutations
SP	CS-1	25.0	99.76%	A23403G, C241T, C884G, G1505A, C3037T, A5852G, G8131T, A8621G, A8833T, T9106C, A9266G, A9656G, A10840G, C13329T, G13690A, C14408T, G18255T, A20755T, G21641A, A21706G, A29735G
	1.2–105	22.0	99.92%	A23403G, C241T, C2455T, C3037T, C6629T, C14408T, C15273T, C17502T, C18877T, C21304T, C21731T, A21917G, G25563T, C25916T, C26735T, T27384C, C28869T, C29095T
	9.8–16	20.0	99.96%	A23403G, C241T, C3037T, C8208T, C14408T, C23987T, C25416T
WP	9.16–21	30.0	99.68%	A23403G, C241T, G2720A, C3037T, C6762T, G7042T, C9226T, C14408T, C16017T, C16111T, T17454A, G19072T, C19216T, A20755T, C21575T, A21706G, C23557T, A23933C, G25429T, G28077T, G28842T, A29301G
	9.17–8	31.0	99.93%	A23403G, C241T, C3037T, C6099A, C6762T, G7042T, C7318A, C9226T, C12854T, C14408T, C14913T, C16017T, C16111T, G19072T, G19997A, C21575T, C23557T, G25429T, G28077T, G28842T
	9.17–11	30.0	99.92%	A23403G, C241T, C3037T, C6762T, G7042T, C9226T, C14408T, C16017T, C16111T, G19072T, C21575T, C23557T, G25429T, G28077T, G28842T

### The application of chip in SARS-COV-2 inspection and quarantine

**Fig. 3:**
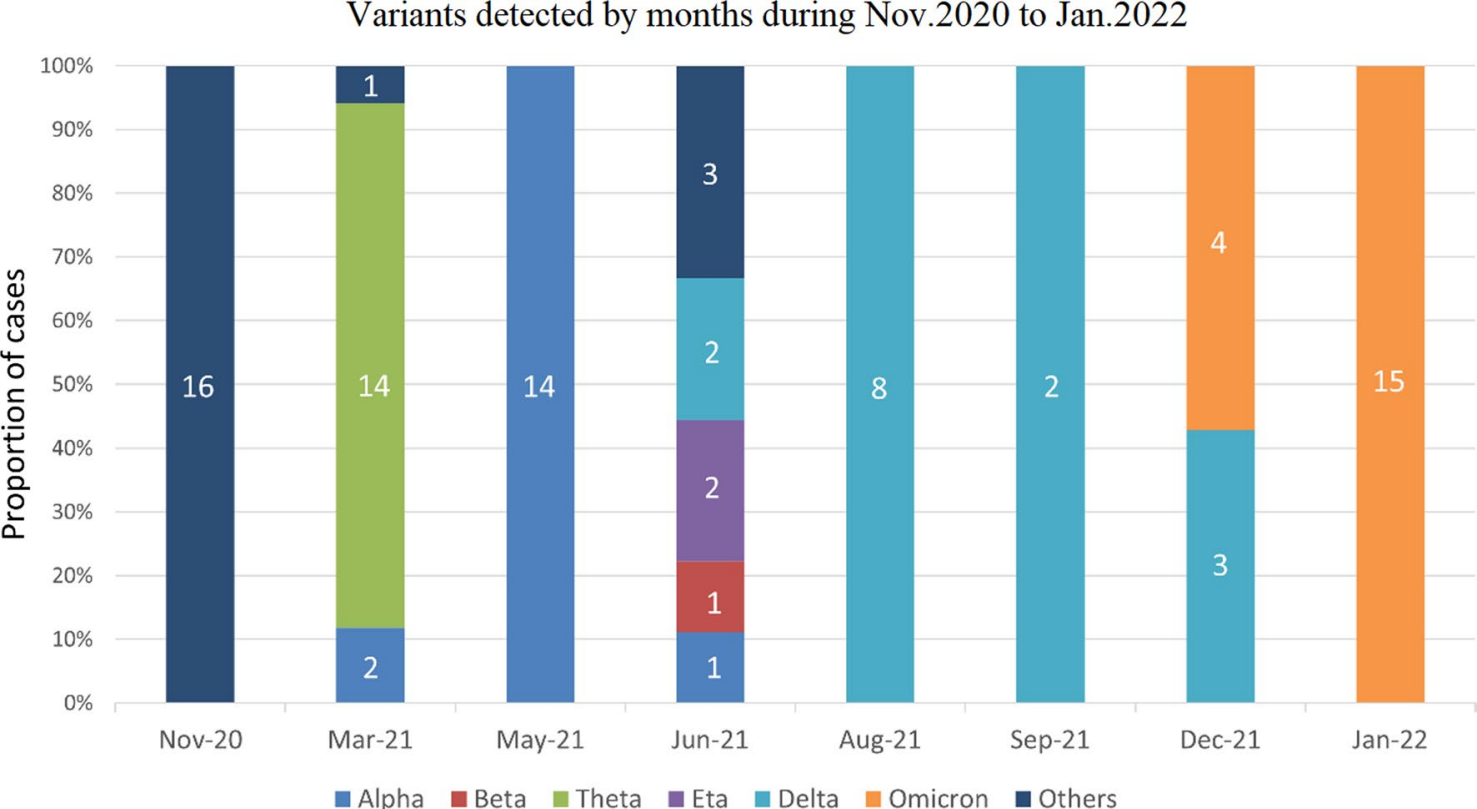
The cumulative number of cases between November 2020 and January 2022 of SARS-CoV-2 variants

With this SARS-CoV-2 genome re-sequencing tiling array, we applied it to the inspection and quarantine at China's entry and exit ports (Hangzhou). From November 2020 to January 2022, Eighty-eight SARS-CoV-2 positive samples (tested by RT-PCR) were sequenced and analyzed. Using the WHO nomenclature system, 4 variants of concern (VOCs) and 2 variants of interest (VOIs) were detected (Fig. 3). Alpha (B.1.1.7) was the first variant declared VOC, it was first discovered in the UK in December 2020. It was identified in 17 cases first detected in March 2021 and predominant in May 2021. Delta (B.1.617.2) was another VOC considered to have increased transmissibility [14]. Totally 15 cases were identified during Q3 and Q4 in 2021 when the Delta variant was observed to have rapidly spread across different continents and taken over dominance [15]. The recently emerged VOC Omicron (B.1.1.529) was identified in December 2021 and quickly reached 100% frequency of 15 tested cases in January 2022. Besides, 1 case of Beta (B.1.351) variant, 16 cases of VOIs including 14 Theta (P.3) variants and 2 Eta (B.1.525) variants, and 20 cases of non-VOC/VOI were primarily identified in early 2021.

## Discussion

Variants can potentially affect the transmission, disease severity, diagnostics, therapeutics, and natural and vaccine-induced immunity [16]. The emergence of highly transmissible SARS-CoV-2 variants of concern (VOCs) such as Delta and Omicron has given rise to massive outbreaks in many countries. It is critical to discriminate against different variants caught at entry and exit ports to support policy decisions. Compared with PCR technology, a full genome tiling array can avoid false negatives caused by mutation occurring at primer binding regions [17]. It can also provide genome sequences and mutations of the virus, which is of great significance for virus traceability, virulence, and vaccine evaluation. Sanger sequencing [18] and Next-generation sequencing (NGS) are mature technology at present. The WHO has recommended that countries sequence at least 1% of their SARS-CoV-2 positive samples to detect emerging VOCs and significant mutations in the virus. Yet, the cost and complexity of workflow make Sanger sequencing and NGS challenging to be widely applied in the front line of anti-epidemic, such as entry and exit ports. For the tiling array, the processing duration from RNA virus to sequencing data is controlled within one day. Whole genome FASTA and FASTQ files are output using customized software based on a bioinformatics protocol of two base calling methods [12]. According to the Sanger sequencing results in this study and the previous reports, this assay accompany the base calling algorithms has ability to discover mutations with a genome-wide accuracy of at least 99.5% [12]. More importantly, this method is highly cost-efficient with $30 per sample and high throughput with 96 parallel testing abilities. Unlike the high-throughput amplicon sequencing[19], barcoding which takes at least 2 h with laborious benchwork is not required for this method. The method in this study is less dependent on equipment and professionals than NGS-based methods. The required equipment includes hybridization ovens, PCR machines, and a chip reader. The operators only need to master the basic operation skills of molecular biology experiments. Of course, like RT-PCR, sequencing results are mostly affected by the viral load in the samples. According to the detection results of clinical samples with known CT values, the coverage of chip sequencing can reach higher than 99% for samples with CT values below 31 so that accurate lineage assignment can be obtained using Pangolin. Furthermore, an updated version of ARTIC multiplex PCR primer sets can improve accuracy at a new emerged variant like omicron [3].

## Data Availability

Not applicable.
